# Editorial: Next-Generation Probiotics: From Commensal Bacteria to Novel Drugs and Food Supplements

**DOI:** 10.3389/fmicb.2019.01973

**Published:** 2019-08-27

**Authors:** Philippe Langella, Francisco Guarner, Rebeca Martín

**Affiliations:** ^1^INRA, Commensal and Probiotics-Host Interactions Laboratory, Micalis Institute, INRA, AgroParisTech, Université Paris-Saclay, Jouy-en-Josas, France; ^2^Digestive System Research Unit, University Hospital Vall d'Hebron, Barcelona, Spain

**Keywords:** probiotic, microbiota, food complement, new drugs, biotherapeutic agent

The concept of traditional probiotics is originally based on the observation that the regular consumption of fermented dairy products with lactic acid bacteria was associated with enhanced health and longevity in elderly Bulgarian people. Since then, the term probiotic has been linked to beneficial bacteria for the host health (Hill et al., 2014). The probiotics field has exploded in the last years due to the increased knowledge of the human gut microbiota and the awareness about health implication of dysbiosis. This new trend highlights that the use of commensal bacteria as probiotics is the natural way to restore a healthy homeostasis situation within the gastrointestinal tract (GIT) opening the door to a new kind of probiotics commonly termed Next-Generation Probiotics (NGP) (Martin and Langella, 2019).

The current Research Topic covers a collection of reviews, mini-reviews, perspectives, opinion, methods, and original research articles focused on the NGPs field. In this sense, two reviews have been focused on the no-scientific concerns. In the first one, El Hage et al. cover current perspectives on the development and assessment of NGPs and the approaches that industry and stakeholders must consider for a successful outcome. In the second one, Brodmann et al. analyze the safety of novel microbes for human consumption with a special focus on the regulatory framework.

Other three non-research articles have been focused on three important NGP candidates. Cani and de Vos wrote a complete state of the art of the well-known beneficial bacterium *Akkermansia municiphila*. Martín et al. focused on the potential bacterial effectors of the multiskilled commensal *Faecalibacterium prausnitzii*. Finally, Rios-Colvian et al. argue about the potential to re-shape the metabolism of *Bacteroides* with specific combinations of dietary carbohydrates-proteins.

Most of the probiotic candidates characterized up today are focused on the GIT. Several research papers published in this topic are focused on the characterization of new strains to be used as probiotic to target GIT related diseases. Nishida et al. describe that *Lactobacillus paraplantarum* 11-1 is able to activate innate immunity and improved survival after infection in silkworm while Bajpai et al. characterize the antibacterial potential of *Pedioccus pentosaceus* 411. Other potential candidates defined on this topic are: *Clostridium butyricum* AQQF01000149 able to block *Salmonella* (Zhao et al.), several strains of *F. praustnizii* characterized from a functional point of view (Martín et al.), isolates of *Enterococcus munditii* that protect insects against *Bacillus thuringiensis* (Grau et al.), and *Bacteroides fragilis* ZY-312 (Wang et al.).

Regarding other ecosystems, Rather et al. have described that *L. sakei* proBio-65 ameliorates the severity of psoriasis-like skin inflammation and López-López et al. reported *Streptococcus dentisani* 7746 and 7747 as an oral probiotic against tooth decay. This strain is able to inhibit the growth of major oral pathogens through the production of bacteriocins, and also buffers acidic pH through an arginolytic pathway. Moving to the respiratory tract, Kanmani et al. described the capacity of the commensal bacterium *Corynebacterium pseudodiphtheriticum* 090104 to improve resistance of infant mice to Respiratory Syncytial Virus and *Streptococcus pneumoniae* superinfection. And finally, hepatorenal damage in broilers has been found to be counterbalanced by *L. plantarum* MYS6 by Deepthi et al.

Several research papers have been focused on the delivery method and/or the food matrix employed to administer the probiotic. Baruzzi et al. describe the development of a symbiotic beverage enriched with bifidobacterial and whey proteins. Navarro et al. present the enhancement of the potential probiotic properties of *L. reuteri* ATCC 23272 when it is delivered as a biofilm on dextranomer microspheres with cargo. Likewise, Dinić et al. introduce the use of post-biotics indicating that *L. fermentum* BGHV110 post-biotic-induced autophagy could be a potential approach for treating acetaminophen hepatoxicity.

Nine original research articles have been focused on the mechanisms of action of several probiotic candidates. Three of them describe the major role of bile salt hydrolases (BSH). Allain et al. in two different manuscripts, describe the anti-giardia activity of *L. johnsonii* La1 *in vitro* and *in vivo* and present BSH as novel target to screen anti-giardia lactobacilli. On the other hand, Rani et al. find by docking analysis that the BSH from *L. gasseri* FR4 could be an inhibitory mechanism to be used as a potential alternative to growth promoters for poultry animals.

Several surface molecules have been described as potential effectors in this topic. Castro-Bravo et al. describe by gene replacement and fluorescent labeling that the different exopolysaccharide of *Bifidobacterium animalis* subsp*. lactis* DSM10140 confer variable functional characteristics to the bifidobacterial surface, which may be relevant for its performance. Besides, the surface protein SlpB form *Propionibacterium freudenreichii* CIRM-BIA 129 has been involved in adhesion to intestinal cells by do Carmo et al. while Veljović et al. found that the aggregation promoting factor AggE of *Enterococcus faecium* BGG09-28 enhances adhesion ability to collagen, mucin, and fibronectin and contribute to the increase of biofilm formation.

Other desirable probiotic characteristic is the ability to positively modulate the immune system. Hidalgo-Cantabrana et al. have combined the use of bioinformatics and *in vitro* tools to screen bioactive peptides encrypted in the human gut metaproteome. Thanks to this strategy, they have identified several peptides able to promote Th17 response in commensal bacteria. Besides, Breyner et al. have found that the microbial anti-inflammatory molecule (MAM) from *F. prausnitzii* is able to inhibit NF-κB pathway protecting mice against several types of colitis.

Selma et al. reported the isolation of human gut bacterial strains that belong to *Eggerthellaceae* family capable of producing isourolithin-A, a urolithin with potential beneficial effects.

Finally, this topic also includes methodological articles, such as the method article of Barone et al. in which a new model of chemically-induced chronic colitis with an outbred murine strain is described to test probiotic candidates. Besides, Arnold et al. have used *L. rhamnosus* AMC143 and AMC010 to demonstrate that the use of both classical microbiology and functional genomics methods are key for the characterization of novel probiotics, as variability between strains can dramatically alter bacterial functionality.

In summary, together the articles of this Research Topic make a substantial contribution to the NGP arena as a step toward a better comprehension of this field.

## Author Contributions

All authors listed have made a substantial, direct and intellectual contribution to the work, and approved it for publication.

### Conflict of Interest Statement

The authors declare that the research was conducted in the absence of any commercial or financial relationships that could be construed as a potential conflict of interest.
